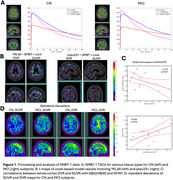# Comparison of dynamic and static properties of a MAO‐B tracer uptake in mild cognitive impairment

**DOI:** 10.1002/alz70855_107376

**Published:** 2025-12-25

**Authors:** Gleb Bezgin, Nesrine Rahmouni, Ryuichi Harada, Nobuyuki Okamura, Andrea L. Benedet, Nicholas J. Ashton, Henrik Zetterberg, Kaj Blennow, Pedro Rosa‐Neto

**Affiliations:** ^1^ Montreal Neurological Institute, Montreal, QC, Canada; ^2^ McGill University, Montreal, QC, Canada; ^3^ Tohoku Medical and Pharmaceutical University, Sendai, Miyagi, Japan; ^4^ Tohoku University Graduate School of Medicine, Sendai, Japan; ^5^ Department of Psychiatry and Neurochemistry, Institute of Neuroscience and Physiology, The Sahlgrenska Academy, University of Gothenburg, Mölndal, Sweden; ^6^ Department of Psychiatry and Neurochemistry, Institute of Neuroscience & Physiology, the Sahlgrenska Academy at the University of Gothenburg, Mölndal, Sweden; ^7^ Clinical Neurochemistry Laboratory, Sahlgrenska University Hospital, Mölndal, Sweden

## Abstract

**Background:**

Astrogliosis is a characteristic feature of the Alzheimer's disease spectrum, commonly manifested by dysregulation of Monoamine Oxidase‐B (MAO‐B). SMBT‐1 is a promising PET tracer for MAO‐B imaging. Here, using a sample of subjects from an AD cohort, we compare results obtained using distribution volume ratio (DVR) and standardized uptake value ratio (SUVR). Using both techniques, we explore the association between these PET metrics and several plasma biomarkers (YKL‐40, Abeta42/40 ratio GFAP, *p*‐tau217 and *p*‐tau231).

**Method:**

SMBT‐1 PET imaging was administered on 22 subjects from the TRIAD cohort (9 cognitively normal (CN), 5 with mild cognitive impairment (MCI), 8 with presumed non‐AD pathology; mean age 66.1). The scanning was performed using High‐Resolution Research Tomograph (HRRT), and the MRI was done on a SIEMENS Prisma scanner. For the PET images, we computed DVR and SUVR, using cerebellar grey as reference region. Proteomics data for plasma GFAP, *p*‐tau‐217, *p*‐tau‐231, Abeta40, Abeta42 and YKL‐40 were obtained using NULISA. The association between DVR/SUVR images and fluid biomarkers was assessed using VoxelStats.

**Result:**

Time activity curves (TACs) for MCI had more sustained tracer retention than those of CN subjects, resulting in generally higher DVR values (Figure 1A). VoxelStats analyses showed that DVR and SUVR were generally consistent in capturing association between PET data and plasma biomarkers (Figure 1B). GFAP, *p*‐tau217 and *p*‐tau231 showed association with voxels around precuneus and medial frontal, whereas YKL‐40 was most associated with periventricular white matter. Abeta42/40 relationship with DV had scattered cortical distribution; this association was moderate and marginally significant (r=‐0.5; *p* = 0.07; Figure 1C, top). GFAP correlated with the whole cortex average of SUVR (r=0.7; *p* <0.05; Figure 1C, bottom). Standard deviation values across subjects suggested greater difference across diagnostic groups using DVR than SUVR (Figure 1D).

**Conclusion:**

Even with a current relatively low powered sample, we saw associations between SMBT‐1 and several AD fluid biomarkers; these associations were consistent between DVR and SUVR, albeit both provided complementary information on diagnostic association. Importantly, this ongoing effort is expected to largely expand this sample which will provide enough power for more advanced statistical analyses and more conclusive observations.